# In this month’s Bulletin

**DOI:** 10.2471/BLT.19.001019

**Published:** 2019-10-01

This month’s special theme is hearing loss: building capacity for a public health response. In editorials, Adrian C Davis & Howard J Hoffman (646) call attention to an increased global prevalence and incidence of hearing loss. Shelly Chadha et al. (647) examine health system requirements for hearing care services.

Gary Humphreys (650–651) reports on efforts to reduce noise exposure in the workplace. Bolajoko Olusanya talks to Sophie Cousins (652–653) about her experience with a late diagnosis of hearing impairment, and her efforts to improve the hearing care provided to children in Nigeria.

## Australia, Brazil, China, Germany, Japan, Netherlands, United Kingdom of Great Britain and Northern Ireland, United States of America 

### Age-related hearing loss and dementia prevention

Michael Yong et al. (699–710) review policies to improve the availability of adults’ hearing aids.

## Bangladesh, India, South Africa and the United States of America 

### Task-shifting to improve access

Jonathan J Suen et al. (681–690) describe community provision of hearing care.

## Malawi

### Tracking outcomes of children diagnosed with hearing impairment

Wakisa Mulwafu et al. (654–662) find substantial loss to follow-up.

## Pacific Island countries and territories

### How far for a hearing test?

Peter R Thorne et al. (719–721) map the provision of hearing care services.

## South Africa

### Checking vision and audition

Susan Eksteen et al. (672–680) use mobile phones to screen preschool children.

## Thailand 

### Screening newborns 

Pittayapon Pitathawatchai et al. (663–671) report on a pilot programme in four hospitals.

## Global

### Seeking global workforce

Mahmood F Bhutta et al. (691–698) estimate needs for primary care providers, audiologists, ear, nose and throat specialists, speech therapists and teachers. 

### Education for children with hearing loss

Joseph J Murray et al. (711–716) explore the contribution of signed languages.

Karissa L LeClair and James E Saunders (722–724) argue for an holistic approach. 

### Audiology apps

De Wet Swanepoel et al. (717–718) uncover strengths and weaknesses of mobile applications.

### Classifications of hearing loss 

Bolajoko O Olusanya et al. (725–728) request a review of terminology. 

